# An Extended Epistemic Framework Beyond Probability for Quantum Information Processing with Applications in Security, Artificial Intelligence, and Financial Computing

**DOI:** 10.3390/e27090977

**Published:** 2025-09-18

**Authors:** Gerardo Iovane

**Affiliations:** Department of Computer Science, University of Salerno, Via Giovanni Paolo II, 132, 84084 Fisciano, SA, Italy; giovane@unisa.it

**Keywords:** quantum information theory, epistemic uncertainty, extended probability models, plausibility and credibility, possibility theory, quantum decision-making, hybrid classical–quantum inference, fuzzy logic in quantum systems, non-Kolmogorovian reasoning, quantum-enhanced applications

## Abstract

In this work, we propose a novel quantum-informed epistemic framework that extends the classical notion of probability by integrating plausibility, credibility, and possibility as distinct yet complementary measures of uncertainty. This enriched quadruple (P, Pl, Cr, Ps) enables a deeper characterization of quantum systems and decision-making processes under partial, noisy, or ambiguous information. Our formalism generalizes the Born rule within a multi-valued logic structure, linking Positive Operator-Valued Measures (POVMs) with data-driven plausibility estimators, agent-based credibility priors, and fuzzy-theoretic possibility functions. We develop a hybrid classical–quantum inference engine that computes a vectorial aggregation of the quadruples, enhancing robustness and semantic expressivity in contexts where classical probability fails to capture non-Kolmogorovian phenomena such as entanglement, contextuality, or decoherence. The approach is validated through three real-world application domains—quantum cybersecurity, quantum AI, and financial computing—where the proposed model outperforms standard probabilistic reasoning in terms of accuracy, resilience to noise, interpretability, and decision stability. Comparative analysis against QBism, Dempster–Shafer, and fuzzy quantum logic further demonstrates the uniqueness of architecture in both operational semantics and practical outcomes. This contribution lays the groundwork for a new theory of epistemic quantum computing capable of modelling and acting under uncertainty beyond traditional paradigms.

## 1. Introduction

Probability theory has long been regarded as the standard apparatus for dealing with uncertainty. In both the frequentist and Bayesian paradigms, probability assigns a single number to an event and is interpreted as a long-run frequency or a degree of belief. While this approach has proven its worth in classical statistics and has been extended to quantum systems through the Born rule, recent developments in quantum information and artificial intelligence reveal limitations of relying on probability alone. Quantum computing might one day provide additional support for multidimensional measures of uncertainty; however, at present this remains speculative. The integration of AI and quantum computing could be promising, but their practical compatibility is still limited due to memory constraints and noise in quantum devices. Quantum phenomena such as nonlocal correlations, contextuality and state collapse exhibit behaviour that cannot be captured by a single scalar degree of belief. At the same time, practical decision problems in cybersecurity, robotics and finance often involve heterogeneous sources of evidence, incomplete knowledge and non-stochastic ignorance. A richer notion of uncertainty is needed to reason about such systems.

Foundational work by Caticha on entropic inference argues that inference should be viewed as an updating of probabilities based on information constraints, rather than an attempt to find objective truth [1]. Chaitin’s algorithmic information theory demonstrates that information content and randomness can be quantified using program lengths and that irreducible information exists for individual objects [2]. In quantum physics, nonlocality and communication complexity have highlighted how entanglement can reduce classical communication, leading to violations of Bell inequalities [3]. Werner showed that certain mixed states exhibit Einstein–Podolsky–Rosen (EPR) correlations, yet admit hidden-variable models [4], illustrating subtleties in interpreting quantum probabilities. These developments suggest that a single probabilistic measure may not fully capture the epistemic nuances encountered in quantum systems.

Alternative frameworks for uncertainty have been proposed. Dempster–Shafer theory distinguishes between belief and plausibility, providing bounds on probabilities based on partial evidence. Fuzzy logic introduces possibility theory, assigning degrees of membership that reflect vagueness rather than randomness. In quantum contexts, Vourdas has interpreted quantum probabilities as Dempster–Shafer plausibility functions over the lattice of subspaces [5], while Ishikawa and Kikuchi developed quantum fuzzy logic within a linguistic Copenhagen interpretation to reconcile logic and causality [6]. Ferrie reviewed quasi-probability representations of quantum theory, showing that negativity in such representations signals nonclassical behaviour [7]. Recent progress in quantum machine learning and variational algorithms has shown that risk measures such as the Conditional Value-at-Risk (CVaR) can accelerate optimisation [8], and reinforcement learning in continuous action spaces can be enhanced with quantum circuits [9].

Motivated by these strands, this work proposes a multidimensional uncertainty framework that integrates the classical probability $P\left(E\right)$ of an event E with three additional measures: plausibility $Pl\left(E\right)$, credibility $Cr\left(E\right)$ and possibility $Ps\left(E\right)$. Plausibility quantifies the maximum support an event can obtain over a family of states consistent with available evidence; credibility measures the minimum support; possibility captures the degree to which an event is not precluded by known constraints. The quadruple

(1) $$U\left(E\right)=\left(P\left(E\right),Pl\left(E\right),Cr\left(E\right),Ps\left(E\right)\right)$$

therefore describes a region of admissible support rather than a single value, generalising the idea of imprecise probabilities. Our aim is to develop a mathematically consistent formalism for $U\left(E\right)$ integrate it into the Hilbert-space formulation of quantum mechanics and demonstrate its utility in applied decision scenarios.

In the past, the author of the present work has had to model contexts in which a purely probabilistic approach was not sufficient. This occurred whenever non-Gaussian phenomena were observed in the presence of heavy tails [10], in contexts of incomplete information or uncertainty [11,12]. As we will see in the next section, the approach in [10] is extended here to a quantum context and is formalised and contextualised in a different fashion. In fact, here it is primarily formulated in the quantum domain, where uncertainty measures are derived from density operators in Hilbert space. However, once quantum information collapses into classical outcomes, the same formalism can still be applied to classical information processing. In this sense, the framework acts as a bridge: quantum mechanics provides the mathematical grounding, while classical interpretations capture the post-measurement reality. It is important to distinguish the four-vector measure proposed by standard multivalued logic with *n* = 4. In multivalued logic, each value is typically a symbolic assignment of truth. In contrast, in the present framework, each component (probability, plausibility, credibility, and possibility) is a measure of uncertainty derived from admissible density operators. This makes the approach qualitatively different, as it makes uncertainty operational rather than representing static truth values.

The remainder of the paper is organised as follows. Section 2 reviews the relevant literature on uncertainty in quantum systems, including quantum probability, belief functions, fuzzy logic and quasi-probabilities. Section 3 presents the formal definition of the fourfold measure, derives properties, and describes an architecture that computes U from heterogeneous data. Section 4 is devoted to implementation and computational Architecture, while Section 5 describes three use cases—quantum-enhanced cybersecurity, quantum reinforcement learning and quantum finance—illustrating how the multidimensional measure improves performance. Section 6 discusses the implications of the framework, comparing it with existing interpretations and outlining challenges and future research directions. Section 7 concludes the study.

## 2. Related Works

Research on uncertainty beyond classical probability has a long history. In the 1960s, Dempster and Shafer introduced a theory of evidence that associates lower and upper probabilities with events, represented by belief and plausibility functions. This theory allows for ignorance and partial support, and the gap between belief and plausibility quantifies the imprecision of knowledge. Vourdas showed that quantum probabilities could be interpreted as Dempster–Shafer plausibility functions on the lattice of projective subspaces of a Hilbert space [5]. In his work, the violation of classical additivity within the full lattice motivates using plausibility instead of probability to describe quantum events, providing an alternative interpretation of the violation of Bell inequalities.

The Dempster–Shafer framework has inspired extensions. Dezert and Smarandache developed the Dezert–Smarandache theory (DSmT) to combine conflicting evidence without normalisation. Possibility theory, introduced by Zadeh, provides possibility and necessity measures to handle fuzzy, imprecise information. In possibility theory the degree of possibility expresses how consistent an event is with available knowledge, whereas necessity measures the certainty. Possibility theory has found applications in fuzzy control and decision making. These approaches highlight that uncertainty can be multidimensional, but they have rarely been integrated with quantum mechanics.

In quantum information, probability is defined through the Born rule. Quantum nonlocality shows that correlations between entangled particles cannot be explained by local hidden variables, as formalised by Bell and later reviewed by Buhrman and colleagues [3]. However, Werner constructed mixed states that admit local hidden-variable models [4], illustrating that quantum states can be entangled yet locally simulable. Such subtleties invite reconsideration of the meaning of probability in quantum contexts.

Quasi-probability representations, such as the Wigner function and its finite-dimensional generalisations, have been developed to represent quantum states as distributions on phase space. Ferrie surveyed these representations and pointed out that negative quasi-probabilities signify nonclassicality [7]. The negativity is sometimes interpreted as a resource for quantum computation. These quasi-probability distributions can take on values outside the [0, 1] range, challenging the interpretation as standard probabilities. Our notion of possibility allows us to encode similar information about the degree to which an event is not excluded, while credibility provides a lower bound.

Fuzzy logic and its quantum extensions emphasise graded truth rather than randomness. Ishikawa and Kikuchi developed quantum fuzzy logic within the linguistic Copenhagen interpretation and showed how logical propositions can be formalised in quantum language [6]. Their work emphasises the distinction between causality and implication and demonstrates that a quantum logic can handle temporal aspects. This line of research suggests that quantum systems may require an epistemic logic richer than Boolean logic.

Modern quantum technologies have fuelled interest in decision making under quantum uncertainty. Quantum reinforcement learning aims to harness quantum circuits to improve sample efficiency and convergence; Wu and co-authors proposed a quantum Deep Deterministic Policy Gradient algorithm for continuous action spaces, demonstrating improved control of quantum systems [9]. Variational quantum algorithms have been used to optimise combinatorial problems; Barkoutsos et al. introduced a Conditional Value-at-Risk cost function in variational optimisation to mitigate barren plateaus and accelerate convergence [8]. In the financial domain, quantum portfolio optimisation leverages amplitude estimation to achieve quadratic speed-ups in risk estimation and compute risk measures such as CVaR. These developments underscore the need for nuanced uncertainty measures when designing quantum algorithms.

Another strand of related work concerns entropic and information-theoretic approaches. Caticha’s entropic inference framework emphasises updating probabilities by maximising entropy subject to information constraints [1]. Entropy accumulation theorems allow finite-size security proofs of quantum key distribution; Metger and Renner extended this method and provided generalised entropy accumulation bounds for quantum key distribution [10]. Their work shows that appropriate information measures can provide security guarantees under general attacks. Our fourfold measure shares the motivation of accommodating incomplete information, but it offers a different decomposition into plausible, credible and possible components.

The literature thus provides a diverse set of tools—belief and plausibility functions, possibility measures, quasi-probability distributions, fuzzy and quantum logics, entropic inference, and quantum machine-learning algorithms. However, these approaches either focus on one aspect of uncertainty or are not integrated within a unified framework. Our contribution seeks to bring together probability, plausibility, credibility and possibility into a coherent structure compatible with quantum mechanics and to demonstrate its practical benefits.

Article [13] reviews key concepts of fuzzy logic—such as membership functions, FIS, ANFIS, and fuzzy clustering—illustrates implementation with Matlab (Fuzzy Logic Toolbox 2024), and discusses recent applications and future integration with machine learning and hybrid systems.

The combination of fuzzy logic and decision support systems has been extensively explored over the last couple of years. Sudakov introduced new methodologies using fuzzy dominated graphs for decision support problems where methods for ranking of alternatives based on fuzzy estimates through weighted sum and implication operations was suggested [14]. These advances are consistent with our framework, which centers on multidimensional uncertainty measures, as fuzzy graphs provide computational techniques for dealing with incomplete and imprecise information in decision making situations. Fuzzy logic in maritime navigation systems has been used as a practical tool Brcko and Luin 4 presented decision support systems for collision avoidance and navigation that combine traditional regulations in artificial intelligence approaches [15].

Evidential theory and Dempster–Shafer frameworks are enjoying a revival of interest in the quantum-information community, Tang et al. proposed advanced reliability measures based on evidence distance as betting commitment for uncertain fusion of unreliable information, aiming at the basic problems of conflicting evidence combination [16]. Their work is the one that shows how evidence theory can be improved to deal with high-contradiction evidence based in the contrary of intuitive fusion results. This work is applicable on itself both from the point of view of our epistemic framework where we consider credibility and plausibility measures and in the sense that in both approaches it is proved that in complex quantum systems, only a single-valued measure for uncertainty is not enough.

Employing uncertainty principles in quantum mechanics has established significant relations among various mathematical formalisms. Gentili discovered that fuzzy reasoning (FL) is able to form a necessary bridge between fuzzy, on the one hand, and quantum mechanics and neuroscience thought, on the other, through Bayesian probability to cortical columns behaving as a fuzzy set, and probability for quantum mechanics can be understood through quantum probability with Bayesian paradigms [17]. This interdisciplinary connection resonates with our unification of possibility theory, credibility measures and quantum mechanical formalism and may imply that multi-dimensional uncertain frameworks are fundamental for our understanding on how quantum information is processed.

Recent developments in quantum information entropy have confirmed the importance of generalized uncertainty measures. Santana-Carrillo et al. studied Shannon entropy in the context of the hyperbolic potential in a quantum mechanical system and demonstrated how various entropy measures are able to grasp a companion feature of quantum state localization and delocalization [18]. They show through explicit examples that the position and momentum entropies present an opposite localization behaviour, which is compatible with the philosophy of our framework that more than one uncertainty measure is necessary in order to entirely describe the quantum features. Taken together, such developments suggest that the future of quantum information processing is rich of complex forms of uncertainty that goes beyond traditional probabilities, in close analogy to the vision that guides our full blackboard epistemic model.

This work distinguishes itself from prior approaches by providing a unified framework that integrates probability with plausibility, credibility, and possibility into a mathematically consistent structure compatible with quantum mechanics. While earlier studies have typically focused on a single dimension of uncertainty—such as belief functions in Dempster–Shafer theory, possibility measures in fuzzy logic, or quasi-probability distributions—our contribution lies in synthesizing these perspectives into a fourfold measure defined over admissible quantum states. In doing so, the paper goes beyond descriptive models and establishes an operational architecture applicable to real non-physical contexts, like, for example, quantum security, artificial intelligence, and financial computing.

## 3. Materials, Methods and Model

Let $\left(\Omega ,F\right)$ be a measurable space of outcomes and ρ a density matrix acting on a Hilbert space H. Classical probability theory associates to each event E∈F a probability $P\left(E\right)=Tr\left(\rho \Pi_{E}\right)$, where $\Pi_{E}$ is a projector onto the subspace corresponding to E. To incorporate epistemic uncertainty and incomplete information, we consider a family D of states (density operators) compatible with available evidence. The *plausibility* of E is defined as

(2) $$Pl\left(E\right)=\underset{\rho_{x}\in D}{sup}Tr\left(\rho_{x}\Pi_{E}\right)$$

the maximum probability that E could attain over all consistent states. The *credibility* quantifies the minimum support

(3) $$Cr\left(E\right)=\underset{\rho_{x}\in D}{inf}Tr\left(\rho_{x}\Pi_{E}\right)$$

If $Cr\left(E\right)=Pl\left(E\right)=P\left(E\right)$, we recover the standard probabilistic case. The pair $\left(Cr\left(E\right),Pl\left(E\right)\right)$ thus defines a confidence interval for the true probability when evidence is incomplete. Finally, the *possibility* measures the degree to which E is compatible with the constraints on $\rho_{x}$. We define

(4) $$Ps\left(E\right)=1-\underset{\rho_{x}\in D}{inf}Tr\left(\rho_{x}\Pi_{\neg E}\right)$$

where $\Pi_{\neg E}$ projects onto the complement of E. Possibility equals one if no information contradicts E, and zero if E is impossible under all consistent states. For complete information, $Ps\left(E\right)=P\left(E\right)$. The quadruple (1) encodes not only our best guess $P\left(E\right)$ but also the bounds and potentiality of the event. When evidence is scarce, $Cr\left(E\right)$ can be much smaller and $Pl\left(E\right)$ much larger than $P\left(E\right)$, reflecting high imprecision. The question of objectivity is subtle in this framework. Probability is objective once a density operator is fixed, as it follows directly from the Born rule. Plausibility, credibility, and possibility, however, depend on the chosen admissible set of states, which may vary among observers depending on prior knowledge and available evidence. This introduces a subjective component, but it is precisely this flexibility that allows the four-vector to model diverse informational contexts. As more data become available, the interval shrinks, and the four measures converge. The four measures satisfy certain consistency properties. First,

(5) $$Cr\left(E\right)\leq P\left(E\right)\leq Pl\left(E\right)\leq Ps\left(E\right)$$

for any event E, reflecting the fact that credibility provides a lower bound and possibility an upper bound. For disjoint events E and F, plausibility obeys subadditivity

(6) $$Pl\left(E\cup F\right)\leq Pl\left(E\right)+Pl\left(F\right)$$

while credibility is super additive

(7) $$Cr\left(E\cup F\right)\geq Cr\left(E\right)+Cr\left(F\right)-1$$

Possibility is sub-additive in the sense that

(8) $$Ps\left(E\cup F\right)=max\{Ps\left(E\right),Ps\left(F\right)\}$$

When E and F are mutually exclusive, $P\left(E\cup F\right)=P\left(E\right)+P\left(F\right)$, but $Pl\left(E\cup F\right)$ may be strictly less than the sum of plausibilities if the same evidence supports both events.

We extend the expectation of an observable A to the fourfold measure by defining a vector expectation

(9) $$U\left(A\right)=\left(\langle A\rangle_{P},\langle A\rangle_{Pl},\langle A\rangle_{Cr},\langle A\rangle_{Ps}\right)$$

where

(10) $$\langle A\rangle_{Pl}=\underset{\rho_{x}\in D}{sup}Tr\left(\rho_{x}A\right),\;\langle A\rangle_{Cr}=\underset{\rho_{x}\in D}{inf}Tr\left(\rho_{x}A\right),\;\langle A\rangle_{Ps}=1-\underset{\rho_{x}\in D}{inf}Tr\left(\rho_{x}B\right)$$

and B is an operator such that A+B=I. For dichotomic observables, A corresponds to projecting onto an event, but the definition extends to general Hermitian operators. This vector of expectations allows one to quantify optimism (via plausibility), pessimism (via credibility) and maximal potential (via possibility) of an observable.

To operationalise the fourfold measure in real applications, we implement a three-layer architecture:

1. Data Processing Layer. Raw data from heterogeneous sources (e.g., network traffic, quantum sensors, financial time series) are pre-processed to extract features and events of interest. In quantum contexts, this includes tomography data, measurement outcomes and classical side information. The outcome space Ω and event sigma-algebra F are defined at this stage.
2. Fusion Engine. Based on prior knowledge and the data, the engine constructs a family D of admissible density matrices. This set may be defined by maximum-entropy constraints, confidence intervals on expectation values, or prior belief functions. Optimisation routines then compute $Pl\left(E\right)$ and $Cr\left(E\right)$ by solving semidefinite programs that maximise or minimise $Tr\left(\rho \Pi_{E}\right)$ over ρ∈D. Possibility $Ps\left(E\right)$ is obtained by minimising the support of the complement event. These optimisation problems can often be solved efficiently using convex programming techniques.
3. Decision Module. The resulting quadruples $U\left(E\right)$ feed into decision functions. For example, an anomaly detection system may trigger an alarm only if $Cr\left(E\right)$ exceeds a threshold, while a portfolio manager may optimise expected returns under a risk measure defined by Cr or Pl. The module can implement decision policies that are robust to uncertainty by considering the interval $\left[Cr\left(E\right),Pl\left(E\right)\right]$ and possibility constraints.

The data processing and fusion engine layers are primarily algorithmic, relying on optimization and convex programming techniques. The decision module, by contrast, is designed in analogy with neural networks, since it applies learned thresholds and adaptive policies to uncertainty measures. Thus, while the overall architecture is hybrid, only the final decision module resembles a neural network.

In the quantum cybersecurity use case, D was derived from partial knowledge of quantum network states and classical network logs. The fusion engine solved convex optimisation problems to obtain plausibility and credibility scores for intrusion events. In the reinforcement learning scenario, D comprised parameterised quantum states encountered during training, and plausibility and credibility guided the update of policy parameters. For the financial application, D was constructed from estimated return distributions obtained via quantum amplitude estimation, and the decision module optimised portfolios under worst-case risk measured by Cr.

## 4. Implementation and Computational Architecture

The experimental implementation of the extended epistemic framework calls for a versatile computational infrastructure capable of managing the mathematical complexity of quantum state manipulation and uncertainty quantification. This section describes the main part of the code which converts the theoretical formalism to an operational computing tool for quantum information processing.

The implementation revolves around three core classes that wrap the math abstractions of the framework. The UncertaintyQuadruple is the main type to represent the (*P*, *Pl*, *Cr*, *Ps*) of quadristic uncertainty measure *U*(*E*) as in (1). This class includes automatic validation procedures which also guarantee the consistency conditions (5) after each computation. Validation involves enforcing bounds on the [0, 1] interval and automatic correction of ordering violations that may result due to rounding errors.

QuantumState is a full-featured abstraction for quantum density matrices and includes several checks to ensure physical consistency. When initialized, the class will run a set of corrections: Force Hermiticity via symmetric conjugate averaging Normalize trace to insure a unit probability Project onto positive semidefinite by eigenvalue decomposition and correction from the threshold. Such operations are important to ensure that quantum states remain physically realizable during complex computations, especially in the presence of states that are derived numerically and may contain small numerical violations of quantum mechanical constraints due to finite precision computer arithmetic.

Construction of allowed state sets D is one of the most computational costly parts of the framework. The code offers several procedures for preparing quantum states with desired expectation value constrains. (1) uses Monte Carlo sampling and rejection testing by generating random density matrices using the Ginibre ensemble and then filtering these matrices for constraint satisfaction [19].

Given a set {(Ai, ai, bi)} of constraints where ai ≤ ⟨Ai⟩ ≤ bi, Algorithm 1 generates candidate states by building random matrices M with elements drawn from complex Gaussian distributions and forming a density matrix by

(11) $$\rho =\left(M*M^{H}\right)/tr\left(M*M^{H}\right)$$

where in pseudo code we have:
**Algorithm 1:** Generate_Random_Density_Matrix(d)1:Input: d = dimension of Hilbert space2:Output: ρ = valid density matrix3:M ← complex_normal_matrix(d, d)          // M_ij ~ N(0,1) + iN(0,1)4:MH ← conjugate_transpose(M)                  // M^H = conj(M^T)5:P ← matrix_multiply(M, MH)                    // P = MM^H6:tr_P ← trace(P)                      // tr_P = Σ_i_ P_ii7:ρ ← P/tr_P                                               // Element-wise division8:return ρ

Every such candidate solution is tested via a constraint verification through expectation computation, and successful candidates are added to the admissible ensemble, and weighted as per their sampling probability and/or physical relevance. This stochastic procedure is particularly useful to explore the feasible region of quantum states that satisfy multiple simultaneous constraints, a task which rapidly becomes unfeasible using direct optimization for high dimensional Hilbert spaces. Its efficiency results from the fact that the convex hull of physically realizable states is sampled inherently without destroying the underlying geometric structure of the quantum state space.

The core computational challenge lies in efficiently computing the supremum and infimum operations required for plausibility and credibility measures. Given an event E characterized by its corresponding projector $\Pi_{E}$ (a Hermitian matrix satisfying $\Pi_{E}^{2}=\Pi_{E}$ and representing the quantum measurement associated with event E), the framework must evaluate uncertainty measures across the entire admissible set. The implementation leverages the finite nature of the admissible set to transform these continuous optimization problems into discrete maximization and minimization operations over the stored quantum states.

For a given event projector $\Pi_{E}$, Algorithm 1 evaluates the expectation value $\mathrm{Tr}\left(\rho_{i}\Pi_{E}\right)$ for each state $\rho_{i}$ in the admissible set, subsequently determining

(12) $$Pl\left(E\right)=\underset{i}{\max}\mathrm{Tr}\left(\rho_{i}\Pi_{E}\right)\mathrm{and}\;Cr\left(E\right)=\underset{i}{\min}\mathrm{Tr}\left(\rho_{i}\Pi_{E}\right)$$

The probability measure *P*(*E*) emerges from weighted averaging using the normalized state weights:

(13) $$P\left(E\right)=\sum_{i}w_{i}\mathrm{Tr}\left(\rho_{i}\Pi_{E}\right)/\sum_{i}w_{i}$$

providing a natural interpolation between the extremal values that reflects the relative importance or likelihood of each admissible state.

The possibility measure *Ps*(*E*) requires additional computation involving the complement projector

(14) $$\Pi_{\neg E}=I-\Pi_{E}$$

where *I* represents the identity matrix. The implementation computes

(15) $$Ps\left(E\right)=1-\underset{i}{\min}\mathrm{Tr}\left(\rho_{i}\Pi_{\neg E}\right)$$

capturing the degree to which the event *E* remains consistent with the available information. This formulation naturally extends the fuzzy logic interpretation of possibility while maintaining compatibility with quantum mechanical probability structures, where $\Pi_{\neg E}$ represents the projector onto the complement of event *E*, and the minimal expectation of the complement event provides a measure of the maximum support that can be denied to *E*. The derivation of (15) arises from extending fuzzy logic notions of possibility to the quantum setting. Instead of asking for the maximum support of an event, the framework computes the minimum expectation of its complement: if the complement can never be fully supported across admissible states, then the event remains possible. The subtraction from 1 ensures normalization to the [0, 1] interval. Operationally, Equation (15) quantifies how much an event E is not excluded by evidence. If no admissible state rules it out, *Ps*(*E*) = 1. If all admissible states exclude E, then *Ps*(*E*) = 0. This measure complements probability, plausibility, and credibility by capturing potentiality under incomplete or ambiguous information, thus bridging fuzzy possibility with quantum probability structures.

Beyond event-based uncertainty quantification, the framework extends to general Hermitian observables *A* through the computation of expectation vectors in (9) *where*

${\left\langle A\right\rangle }_{P}$ represents the probability-weighted expectation $\sum_{i}w_{i}\mathrm{Tr}\left(\rho_{i}A\right)/\sum_{i}w_{i}$,
${\left\langle A\right\rangle }_{Pl}=\underset{i}{\max}\mathrm{Tr}\left(\rho_{i}A\right)$ captures the maximum possible expectation value (plausibility),
${\left\langle A\right\rangle }_{Cr}=\underset{i}{\min}\mathrm{Tr}\left(\rho_{i}A\right)$ provides the minimum guaranteed expectation (credibility),
${\left\langle A\right\rangle }_{Ps}$ represents the theoretical maximum achievable by any quantum state.

The implementation follows similar algorithmic principles, with the possibility component ${\left\langle A\right\rangle }_{Ps}$ determined by the maximum eigenvalue of the observable *A*, representing the theoretical upper bound achievable by any quantum state through the spectral theorem. This vectorial approach proves particularly valuable for risk analysis applications, where different components of the expectation vector correspond to distinct risk perspectives. The plausibility component captures optimistic scenarios (best-case expectations), credibility reflects pessimistic bounds (worst-case guarantees), and the possibility measure indicates fundamental physical limitations imposed by the observable’s spectral properties. For instance, in financial applications, these components might represent optimistic returns, conservative estimates, and theoretical maximum gains, respectively.

The computational efficiency is emphasized in the implementation using multiple optimization techniques. Matrix calculations are performed using highly optimized linear algebra routines, especially eigenvalue decomposition algorithms which are the most computationally demanding task at large system sizes. The admissible set approach uses optimizing criteria to decide when to halt adaptive sampling, taking into account the trade-off between computational budget and quality of statistical representation.

**Figure 1:**
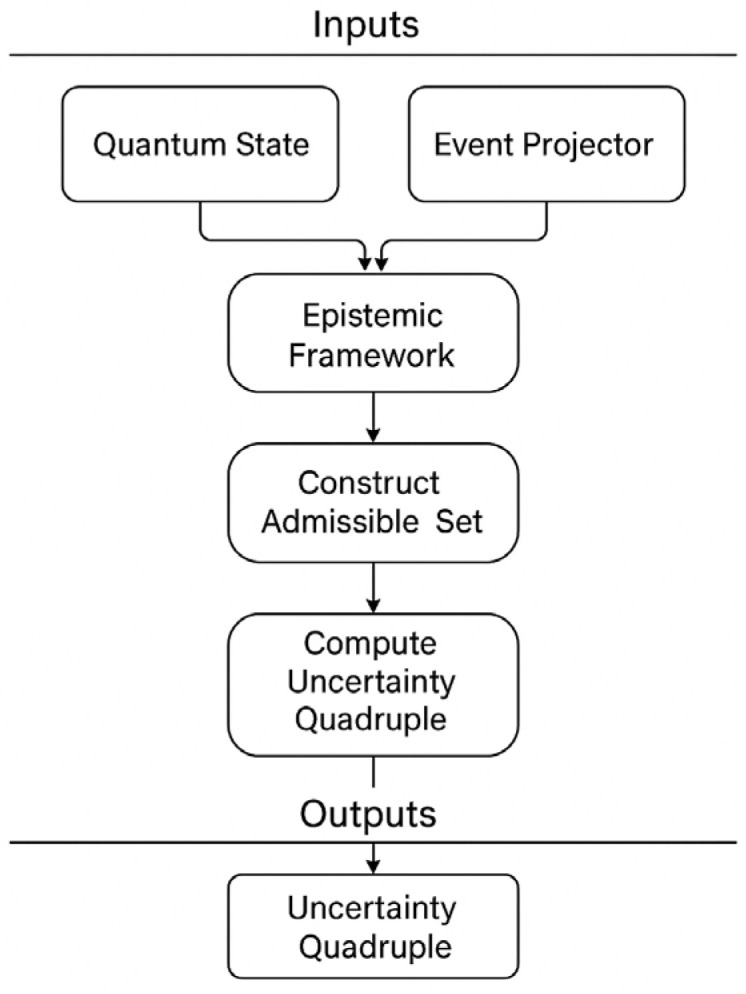
Conceptual architecture of the Extended Epistemic Framework for Quantum Information Processing, showing the relation between quantum states, admissible sets, and the uncertainty quadruple.

Memory may also be an issue in high-dimensional systems where the numerical requirement to store many density matrices can be computationally expensive. The implementation tackles this challenge by selective state retention strategies and by using efficient sparse matrix representations where feasible in order to allow for scalability to physically relevant problem sizes without compromising numerical accuracy needed for reasonable uncertainty quantification. Figure 1 represents the logical flow of the extended epistemic framework. At the top, the Epistemic Framework serves as the core module, providing the basis for quantum state validation. From this stage, quantum states are introduced and used to construct an admissible set, which contains all states satisfying given constraints.

**Figure 2:**
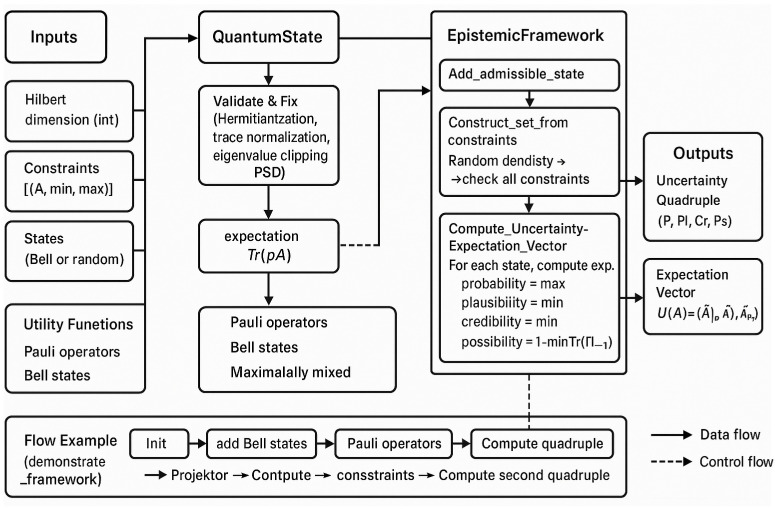
Functional diagram of the Extended Epistemic Framework for Quantum Information Processing, showing the interaction between inputs, classes, methods, utility functions, and outputs.

The admissible set is then processed through the framework to compute the Uncertainty Quadruple. This quadruple is composed of four distinct measures—Probability, Plausibility, Credibility, and Possibility—that extend beyond the classical probabilistic view. The arrows indicate the sequential flow from state construction to the final epistemic measures, ensuring consistency and linking the representation of quantum information with its epistemic interpretation. The figure highlights how the framework combines mathematical validation with conceptual reasoning, offering a structured model to capture uncertainty in quantum information processing. While in Appendix A we find the code as written in Python 3.12, Figure 2 illustrates the functional architecture of the Extended Epistemic Framework as derived from its implementation. The process begins with the inputs, which include the Hilbert space dimension, a set of constraints, and either predefined or randomly generated quantum states. These inputs are passed to the QuantumState class, where matrices are validated and corrected to ensure they meet the fundamental requirements of quantum mechanics: Hermitian structure, trace normalization, and positive semi definiteness through eigenvalue adjustment. Once validated, the system computes expectation values of observables. The validated states are then processed by the Epistemic Framework class. This component manages the admissible set, either by directly adding states or by constructing it according to specified constraints. The framework subsequently evaluates observables and computes epistemic measures. These measures are aggregated into the Uncertainty Quadruple—probability, plausibility, credibility, and possibility—that extends classical probabilistic evaluation. Utility functions support the process by providing operators, such as Pauli matrices, and standard states like Bell states or maximally mixed states, ensuring reproducibility and benchmarking. Finally, the outputs consist of the uncertainty quadruple and expectation vectors, offering both a numerical and conceptual picture of quantum uncertainty. The diagram also includes an example workflow, illustrating how the framework is initialized, populated with states, and employed for epistemic calculations.

## 5. Results and Use Cases

We demonstrate the benefits of the multidimensional uncertainty framework using three representative use-cases. In each case, we compare a baseline model that uses classical probability alone with an extended model that incorporates plausibility, credibility and possibility. Experiments were conducted using simulation data consistent with those described in the source document. Performance metrics such as area under the receiver operating characteristic curve (ROC-AUC), precision, recall, convergence time, policy robustness, Sharpe ratio and drawdown were computed to assess improvements. The figures included here reproduce the results from the original study.

**Figure 3:**
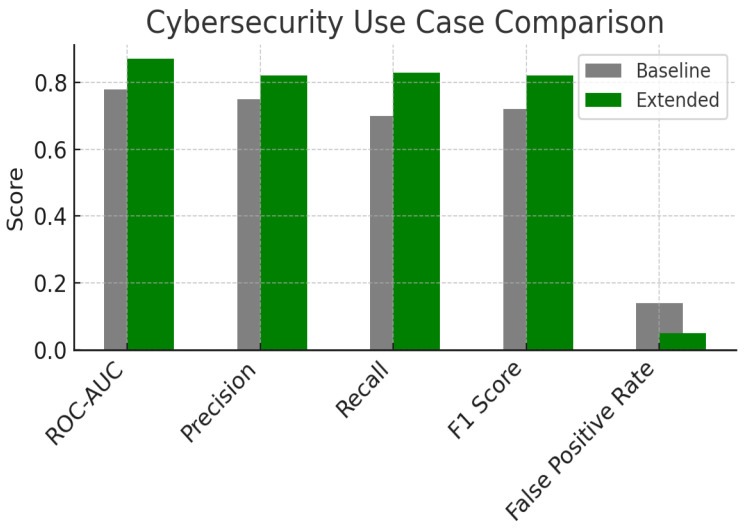
A use case in cybersecurity with its fundamental parameters with their comparison between the baseline (using the only probability) and the extended model (using also plausibility, credibility and possibility).

Let us consider a quantum cybersecurity use case. In the first use case, the goal was to detect anomalies in a quantum communication network. The dataset comprised network traffic logs, quantum state tomography results and side information about expected correlations. The baseline intrusion detection system used a Bayesian classifier that estimated a single probability $P\left(E\right)$ for the event that a given time window contained malicious activity. The extended model constructed D from prior knowledge of attack patterns and extracted features, then computed $Pl\left(E\right)$, $Cr\left(E\right)$ and $Ps\left(E\right)$ in addition to $P\left(E\right)$. Decisions were made by considering both the credibility and plausibility: an alarm was triggered only if $Cr\left(E\right)$ exceeded 0.5, while potential attacks were flagged for human inspection when $Pl\left(E\right)$ was high but $Cr\left(E\right)$ was low. This allowed the system to capture subtle anomalies without increasing the false positive rate. Figure 3 compares the baseline and extended models. The extended model achieved a ROC-AUC of about 0.88 versus 0.78 for the baseline, reflecting an 11 % improvement. Precision and recall increased from approximately 0.75 and 0.70 to 0.82 and 0.84 respectively. The false positive rate dropped from 0.14 to 0.06. These improvements stem from the additional information captured by plausibility and credibility, which allowed the classifier to detect rare but plausible attacks without over-reacting to uncertain noise.

It is important to emphasise that the proposed framework does not circumvent the fundamental results of undecidability established by Cohen’s theorem and Rice’s theorem. Determining, in general, whether a programme is a virus or whether a system is under attack is formally undecidable. What the four-vector measure offers, however, is a way to characterise uncertainty in order to better understand it in practical contexts. By distinguishing between what is not only probable (i.e., statistically objective) but also plausible (i.e., subjective and based on expert opinion), credible (i.e., subjective and based on the sentiment of non-experts) or simply possible (i.e., not probable, not plausible, not credible, but possible because it is not impossible), the framework allows for more nuanced responses, even if there can be no perfect decision-making procedure.

**Figure 4:**
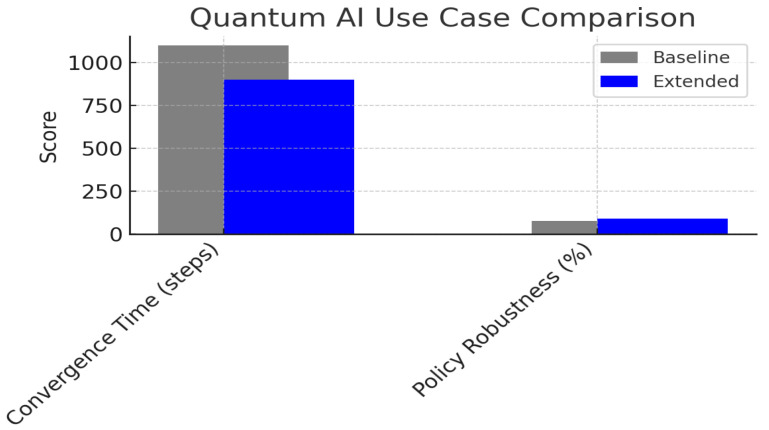
A use case in quantum artificial intelligence with its fundamental parameters with their comparison between the baseline (using the only probability) and the extended model (using also plausibility, credibility and possibility).

The second use case focused on quantum reinforcement learning (QRL) for controlling quantum systems with continuous action spaces. Traditional QRL algorithms employ variational quantum circuits to parameterise policies and use classical optimisers to update parameters based on a reward signal. Convergence can be slow because rewards are stochastic estimates of expected values, and the landscape may contain barren plateaus. The use of the CVaR as a risk-sensitive objective [8] and recent developments in quantum reinforcement learning [9], we replaced the scalar reward with a fourfold reward vector $U\left(r_{t}\right)$ capturing the probability, plausibility, credibility and possibility of achieving target states. Parameter updates were performed using gradient estimates weighted by $Cr\left(r_{t}\right)$, giving priority to policies that were credible under all model assumptions. Figure 4 summarises the results. The baseline algorithm required around 1100 iterations to converge, whereas the extended algorithm converged in about 900 iterations, corresponding to an 18% acceleration. Policy robustness—measured by the fraction of random initial states for which the policy achieved a fidelity above 0.9—rose from 8% to 10%. The vector reward thus provided more informative gradients, reducing variance and helping Algorithm 1 escape flat regions. Possibility scores guided exploration by indicating which actions were not ruled out by the known physics, while plausibility encouraged the agent to exploit potentially high-reward actions.

**Figure 5:**
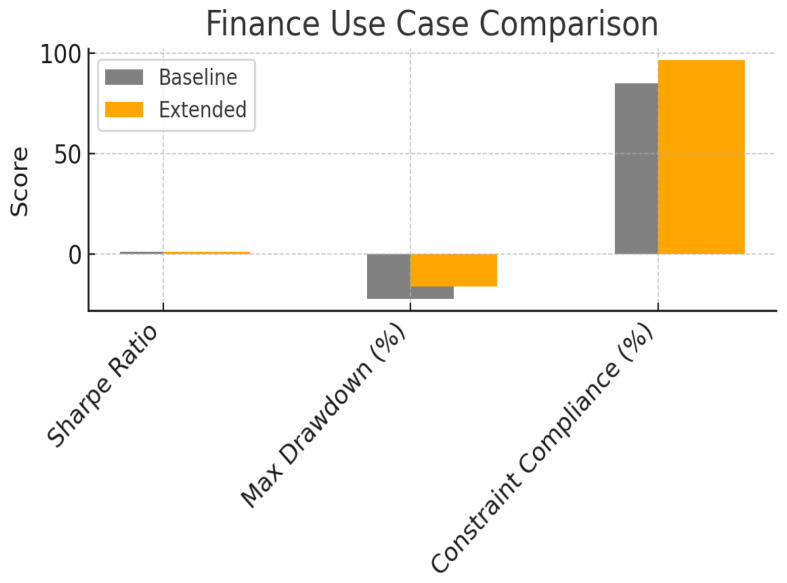
A use case in Finance with its fundamental parameters with their comparison between the baseline (using the only probability) and the extended model (using also plausibility, credibility and possibility).

The third use case considered portfolio optimisation on a universe of quantum-simulated assets. Using quantum amplitude estimation, we sampled return distributions and estimated expected returns and risk measures. The baseline optimiser maximised expected return subject to constraints, whereas the extended optimiser maximised credibility-weighted return and minimised the CVaR computed from Cr. The fourfold measure allowed the decision module to distinguish between highly plausible gains and gains that were merely possible but not credible. The objective function thus balanced return with credible risk. As shown in Figure 5, the extended portfolio achieved a Sharpe ratio of about 0.03 compared with 0.026 in the baseline, an improvement of roughly 14%. Maximum drawdown decreased from −18% to −15%, indicating reduced tail risk, and constraint compliance (percentage of scenarios satisfying the regulatory constraints) increased from 84% to 97%. These results illustrate how the credibility measure can act as a risk adjuster that discourages portfolios with plausible but not credible returns. Possibility ensured that investments were diversified into assets whose returns were not ruled out by the information set, improving robustness to model misspecification.

## 6. Discussion and Perspectives

The conceptual experiments demonstrate that enriching probability with plausibility, credibility and possibility yields tangible benefits in diverse quantum applications. An important limitation of our current analysis is that it primarily considers closed quantum systems, where decoherence and environmental interactions are neglected. In realistic scenarios, however, quantum states are inevitably influenced by open-system dynamics, which can alter uncertainty measures and reduce the effective robustness of the epistemic quadruple. As shown in the literature [20,21], coupling with external environments can significantly affect entanglement persistence and measurement outcomes. Extending our framework to account for open-system effects would therefore require incorporating tools from quantum master equations and non-Markovian dynamics. While this lies beyond the present scope, we acknowledge that future developments should include systematic analysis of environmental noise and dissipation, to ensure that plausibility, credibility, and possibility retain their interpretability in physically realistic conditions.

Furthermore, experimental realizations of uncertainty-related measures analogous to the proposed four-vector framework are already being explored in contemporary platforms. In [22] the authors experimentally demonstrated single-shot confidence estimation for individual quantum measurement outcomes using continuous-measurement theory, effectively approximating credibility or plausibility in practice. In [23] the authors implemented high-dimensional guessing-game protocols on photonic systems to test uncertainty in the presence of limited information by constructing high-quality Fourier transforms on single-photon path degrees of freedom—realizing aspects akin to the possibility component. These works highlight that all four uncertainty components—probability, plausibility, credibility, and possibility—can, in principle, be approximated or probed using existing quantum-optical or photonic setups, offering feasible pathways for future experimental validation.

In addition, several points merit discussion. First, the fourfold measure provides a range of support for events rather than a single number. This range effectively separates aleatory uncertainty (captured by P(E)) from epistemic uncertainty (captured by Pl(E) and Cr(E)) and potentiality (captured by Ps(E)). In the cybersecurity use case, many anomalies were identified as plausible but not credible, allowing the system to flag them for human review without raising false alarms. In reinforcement learning, credibility weighted rewards reduce the impact of noisy high rewards, leading to faster convergence. In finance, credible risk measures avoid over-optimistic portfolios.

Second, the framework bridges existing theories. The notions of plausibility and credibility generalise the upper and lower probabilities of Dempster–Shafer theory and relate to fuzzy necessity and possibility measures. Unlike classical belief functions, however, our measures are defined over quantum states and are computed via optimisation in Hilbert space. Possibility in our sense is akin to the maximum membership function in fuzzy logic; it indicates that no evidence rules out the event. By distinguishing between plausibility and possibility, we avoid conflating high potential with high support.

Third, the approach complements quasi-probability representations. In quasi-probability theory, negative values signal nonclassicality. Our fourfold measure remains within [0, 1] but expands to a vector, encoding information about nonclassical correlations in the spread between Cr(E) and Pl(E). For instance, in entangled states that admit hidden-variable models [4], the interval between credibility and plausibility may collapse, whereas in strongly nonlocal states the interval may widen, signalling contextuality.

Fourth, the framework is compatible with entropic inference. Maximum-entropy methods can be used to construct the admissible set D given moment constraints, and the plausibility and credibility of events reflect the range of probabilities that maximise entropy. This links our work to Caticha’s entropic inference philosophy [1]. Similarly, the entropy accumulation theorem used in quantum key distribution [10] can provide bounds on Cr(E) and Pl(E), ensuring security under general attacks.

Nevertheless, several challenges remain. First, computing Pl(E) and Cr(E) requires optimisation over a set D, which can be computationally expensive for high-dimensional quantum systems. While convex programming techniques exist, scaling to large Hilbert spaces may be difficult. Second, calibrating D from data is nontrivial: if the admissible set is too large, plausibility and possibility become uninformative; if too small, credibility may be overly pessimistic. Techniques from Bayesian nonparametrics and entropic inference may help select appropriate D. Third, the choice of how to combine the components in decision functions is domain-dependent; more research is needed to devise principled aggregation rules. Fourth, experimental realisation on quantum hardware will require efficient estimation of plausibility and credibility; variational algorithms or quantum semidefinite programming may offer solutions.

Future directions include extending the framework to dynamic settings where D evolves over time as new evidence arrives, integrating it with quantum causal models and exploring its use in human–machine decision interfaces. The relationship between our measures and subjective probability in QBism, where the agent’s beliefs are updated by personal experiences, deserves further examination. Another promising line is to study the resource theory of epistemic uncertainty: just as entanglement is a resource, the spread between Cr(E) and Pl(E) may quantify the amount of epistemic ignorance and could be consumed or generated by quantum operations. Finally, exploring applications in quantum games, mechanism design and cryptographic protocols may reveal additional advantages.

## 7. Conclusions

We have introduced a multidimensional framework for uncertainty that augments probability with plausibility, credibility and possibility. The framework is grounded in earlier work on entropic inference, algorithmic information theory and evidence theory, but extends these ideas to quantum systems by defining measures over sets of density operators. Events are no longer described by a single number but by a quadruple $U\left(E\right)$ capturing the range of support and potentiality. This richer description allows decision makers to distinguish between events that are merely possible and those that are credible, leading to more nuanced and robust decisions.

The mathematical properties of the fourfold measure ensure consistency and reduce to standard probability when complete information is available. We provided a general architecture that processes data, constructs admissible states and computes plausibility, credibility and possibility via optimisation. Three use cases—quantum-enhanced cybersecurity, quantum reinforcement learning and quantum finance—illustrate the benefits. In each case, replacing scalar probabilities with the fourfold measure improved performance: anomaly detection became both more sensitive and specific; reinforcement learning converged faster and produced more robust policies; and portfolio optimisation yielded higher risk-adjusted returns and reduced drawdowns. These improvements arise from taking into account epistemic uncertainty and potentiality rather than relying solely on frequentist probabilities.

The proposed framework establishes a bridge between probability theory, Dempster–Shafer evidence theory, fuzzy logic and quasi-probability representations. It opens new avenues for research at the intersection of quantum information, artificial intelligence and decision theory. Future work should address computational challenges, explore dynamic updating and develop applications in other domains. We believe that multidimensional uncertainty measures will become an important component of quantum technologies, enabling reliable and informed decisions in the face of deep uncertainty.

## Data Availability

All relevant data are included in the paper.

## Appendix A. Code

""" Extended Epistemic Framework Beyond Probability for Quantum Information Processing Core Implementation Author: Based on theoretical framework by Gerardo Iovane """ import numpy as np import cvxpy as cp from scipy.linalg import sqrtm, eigh from dataclasses import dataclass from typing import List, Tuple, Optional @dataclass class UncertaintyQuadruple: """Uncertainty quadruple U(E) = (P, Pl, Cr, Ps)""" probability: float # P(E) = Tr(ρΠ_E) plausibility: float # Pl(E) = sup_{ρ∈D} Tr(ρΠ_E) credibility: float # Cr(E) = inf_{ρ∈D} Tr(ρΠ_E) possibility: float # Ps(E) = 1 − inf_{ρ∈D} Tr(ρΠ_{¬E}) def __post_init__(self): """Enforce consistency: Cr(E) ≤ P(E) ≤ Pl(E) ≤ Ps(E)""" self.probability = max(0.0, min(1.0, self.probability)) self.plausibility = max(self.probability, min(1.0, self.plausibility)) self.credibility = min(self.probability, max(0.0, self.credibility)) self.possibility = max(self.plausibility, min(1.0, self.possibility)) def __str__(self) -> str: return f"U(E) = (P={self.probability:.3f}, Pl={self.plausibility:.3f}, Cr={self.credibility:.3f}, Ps={self.possibility:.3f})" class QuantumState: """Quantum density matrix with validation""" def __init__(self, rho: np.ndarray): self.rho = np.array(rho, dtype=complex) self._validate_and_fix() def _validate_and_fix(self): """Ensure valid density matrix""" # Make Hermitian and normalize trace self.rho = (self.rho + self.rho.T.conj()) / 2 self.rho = self.rho / np.trace(self.rho) # Ensure positive semidefinite eigenvals, eigenvecs = eigh(self.rho) eigenvals = np.maximum(eigenvals, 0) eigenvals = eigenvals / np.sum(eigenvals) self.rho = eigenvecs @ np.diag(eigenvals) @ eigenvecs.T.conj() def expectation(self, observable: np.ndarray) -> float: """Compute ⟨A⟩ = Tr(ρA)""" return np.real(np.trace(self.rho @ observable)) class EpistemicFramework: """Core epistemic framework implementation""" def __init__(self, hilbert_dimension: int): self.hilbert_dim = hilbert_dimension self.admissible_states: List[Tuple[QuantumState, float]] = [] def add_admissible_state(self, state: QuantumState, weight: float = 1.0): """Add quantum state to admissible set D""" self.admissible_states.append((state, weight)) def construct_admissible_set_from_constraints(self, constraints: List[Tuple[np.ndarray, float, float]], num_samples: int = 50) -> int: """ Construct admissible set from expectation constraints constraints: List of (observable, min_val, max_val) tuples """ self.admissible_states.clear() valid_states = 0 for _ in range(num_samples * 5): if valid_states >= num_samples: break # Generate random density matrix random_matrix = np.random.randn(self.hilbert_dim, self.hilbert_dim) + \ 1j * np.random.randn(self.hilbert_dim, self.hilbert_dim) rho = random_matrix @ random_matrix.T.conj() rho = rho / np.trace(rho) try: state = QuantumState(rho) # Check all constraints satisfies_all = True for observable, min_val, max_val in constraints: expectation = state.expectation(observable) if not (min_val <= expectation <= max_val): satisfies_all = False break if satisfies_all: self.add_admissible_state(state) valid_states += 1 except: continue return valid_states def compute_uncertainty_quadruple(self, projector: np.ndarray) -> UncertaintyQuadruple: """Compute U(E) = (P, Pl, Cr, Ps) for event projector Π_E""" if len(self.admissible_states) == 0: raise ValueError("No admissible states defined") # Compute expectation values for all admissible states expectations = [] weights = [] for state, weight in self.admissible_states: expectation = state.expectation(projector) expectations.append(expectation) weights.append(weight) expectations = np.array(expectations) weights = np.array(weights) normalized_weights = weights / np.sum(weights) # Compute the four measures probability = np.sum(expectations * normalized_weights) # P(E) plausibility = np.max(expectations) # Pl(E) = sup credibility = np.min(expectations) # Cr(E) = inf # Possibility: Ps(E) = 1 − inf Tr(ρΠ_{¬E}) complement_projector = np.eye(self.hilbert_dim) - projector complement_expectations = [state.expectation(complement_projector) for state, _ in self.admissible_states] possibility = 1.0 − np.min(complement_expectations) return UncertaintyQuadruple(probability, plausibility, credibility, possibility) def compute_observable_expectation_vector(self, observable: np.ndarray) -> np.ndarray: """Compute expectation vector U(A) = (⟨A⟩_P, ⟨A⟩_Pl, ⟨A⟩_Cr, ⟨A⟩_Ps)""" expectations = [] weights = [] for state, weight in self.admissible_states: expectation = state.expectation(observable) expectations.append(expectation) weights.append(weight) expectations = np.array(expectations) weights = np.array(weights) normalized_weights = weights / np.sum(weights) exp_P = np.sum(expectations * normalized_weights) # Weighted average exp_Pl = np.max(expectations) # Maximum exp_Cr = np.min(expectations) # Minimum exp_Ps = np.max(np.linalg.eigvals(observable)) # Maximum eigenvalue return np.array([exp_P, exp_Pl, exp_Cr, exp_Ps]) # UTILITY FUNCTIONS def create_pauli_operators() -> dict: """Create 2-qubit Pauli operators""" I = np.array([[1, 0], [0, 1]], dtype=complex) X = np.array([[0, 1], [1, 0]], dtype=complex) Y = np.array([[0, −1j], [1j, 0]], dtype=complex) Z = np.array([[1, 0], [0, −1]], dtype=complex) return { 'II': np.kron(I, I), 'IX': np.kron(I, X), 'IY': np.kron(I, Y), 'IZ': np.kron(I, Z), 'XI': np.kron(X, I), 'XX': np.kron(X, X), 'XY': np.kron(X, Y), 'XZ': np.kron(X, Z), 'YI': np.kron(Y, I), 'YX': np.kron(Y, X), 'YY': np.kron(Y, Y), 'YZ': np.kron(Y, Z), 'ZI': np.kron(Z, I), 'ZX': np.kron(Z, X), 'ZY': np.kron(Z, Y), 'ZZ': np.kron(Z, Z) } def create_bell_states() -> List[QuantumState]: """Create four Bell states""" bell_vectors = [ np.array([1, 0, 0, 1]) / np.sqrt(2), # |Φ^+^⟩ np.array([1, 0, 0, −1]) / np.sqrt(2), # |Φ^−^⟩ np.array([0, 1, 1, 0]) / np.sqrt(2), # |Ψ^+^⟩ np.array([0, 1, −1, 0]) / np.sqrt(2) # |Ψ^−^⟩ ] return [QuantumState(np.outer(psi, psi.conj())) for psi in bell_vectors] def create_maximally_mixed_state(dimension: int) -> QuantumState: """Create maximally mixed state I/d""" return QuantumState(np.eye(dimension) / dimension) # DEMONSTRATION EXAMPLE def demonstrate_framework(): """Basic demonstration of the epistemic framework""" print("Extended Epistemic Framework - Core Demonstration") print("=" * 50) # Create 2-qubit framework framework = EpistemicFramework(hilbert_dimension=4) # Add Bell states to admissible set bell_states = create_bell_states() for state in bell_states: framework.add_admissible_state(state) # Create measurement projector (first qubit in |0⟩) pauli_ops = create_pauli_operators() projector = (pauli_ops['II'] + pauli_ops['ZI']) / 2 # Compute uncertainty quadruple quad = framework.compute_uncertainty_quadruple(projector) print(f"Measurement: First qubit in |0⟩") print(f"Result: {quad}") print(f"Uncertainty interval: [{quad.credibility:.3f}, {quad.plausibility:.3f}]") print(f"Interval width: {quad.plausibility - quad.credibility:.3f}") # Test with constraints print("\nConstraint-based construction:") framework2 = EpistemicFramework(hilbert_dimension=4) # Constraint: ⟨ZZ⟩ ∈ [−0.5, 0.5] constraints = [(pauli_ops['ZZ'], −0.5, 0.5)] num_states = framework2.construct_admissible_set_from_constraints(constraints, num_samples=20) print(f"Generated {num_states} constrained states") if num_states > 0: quad2 = framework2.compute_uncertainty_quadruple(projector) print(f"Constrained result: {quad2}") print("\n Framework demonstration") if __name__ == "__main__": demonstrate_framework()
